# Berberine Ameliorates Diabetes-Associated Cognitive Decline through Modulation of Aberrant Inflammation Response and Insulin Signaling Pathway in DM Rats

**DOI:** 10.3389/fphar.2017.00334

**Published:** 2017-06-06

**Authors:** Qingjie Chen, Ran Mo, Ninghua Wu, Xin Zou, Cai Shi, Jing Gong, Jingbin Li, Ke Fang, Dingkun Wang, Deshen Yang, Kaifu Wang, Juan Chen

**Affiliations:** ^1^Institute of Integrated Traditional Chinese and Western Medicine, Tongji Hospital, Tongji Medical College, Huazhong University of Science and TechnologyWuhan, China; ^2^Department of Biochemistry and Molecular Biology, School of Basic Medicine and the Collaborative Innovation Center for Brain Science, Tongji Medical College, Huazhong University of Science and TechnologyWuhan, China; ^3^Hubei Key Laboratory of Cardiovascular, Cerebrovascular, and Metabolic Disorders, Hubei University of Science and TechnologyXianning, China; ^4^Department of Integrated Traditional Chinese and Western Medicine, Tongji Hospital, Tongji Medical College, Huazhong University of Science and TechnologyWuhan, China; ^5^Institute for Brain Research, Huazhong University of Science and TechnologyWuhan, China

**Keywords:** berberine, cognitive, inflammation, insulin, medial prefrontal cortex, neuron and astrocyte, type 2 diabetes mellitus, Aβ

## Abstract

**Background:** Memory-impairment was one of the common characteristics in patients with diabetes mellitus. The release of chronic inflammation mediators and insulin resistance in diabetic brain gave rise to the generation of toxic factor Aβ42 which was the marker of Alzheimer’s disease. In addition, the impairment of memory in diabetes mellitus was also correlated predominantly with uptake/metabolism of glucose in medial prefrontal cortex (mPFC). Previously, anti-inflammation and hypoglycemic effects of berberine (BBr) have been described in peripheral tissues. For better understanding the effects of BBr on cognitive action in diabetics, we investigated the functions of BBr involved in anti-inflammation and ameliorating insulin resistance in prefrontal cortex of diabetic rats.

**Methods:** Intragastric administration of BBr (187.5 mg/Kg/d) was used in diabetic rats. Fear-condition assay was applied for cognitive assessment, and relative protein expressions were detected by western-blot. The glucose uptake in prefrontal cortex of diabetic rats was tested by Positron-Emission Tomography imaging. The levels of inflammation mediators were determined by commercial ELISA kits.

**Results:** The inflammation mediator release and insulin resistance in the mPFC of diabetic rats was inhibited by BBr. The activation of PI3K/Akt/mTOR and MAPK signaling pathway, as well as two novel isoforms PKCη and PKC𝜀 and the translocation of NF-κB in neuron were also down-regulated by BBr; furthermore, the neuron specific glucose transporter GLUT3 was remarkably augmented by 2–3 times when compared with diabetic group; meanwhile, BBr also promoted glucose uptake in the brain. Additionally BBr decreased the expressions of amyloid precursor protein and BACE-1, and the production of oligomeric Aβ42. Finally, it accelerates the reinforcement of the information and ameliorates cognitive impairment.

**Conclusion:** BBr inhibited the activation of inflammation pathway and insulin resistance in the mPFC of diabetic rats. Finally, it improved the lesion of cognition in diabetic rats.

## Introduction

Type 2 diabetes mellitus (T2DM) is a chronic and common metabolic disease with a worldwide increase in prevalence, and is associated with severe syndrome and characterized with deficit in insulin production or sensitivity (Costa et al., 2004). Large-population investigations have revealed that diabetes mellitus is associated with Alzheimer’s disease (AD) (Gasparini et al., 2002), mild cognitive impairment (MCI) (Li W. et al., 2016), anxiety and depression (Anderson et al., 2001) in Central Nervous system. AD is even considered as type 3 diabetes mellitus due to insulin resistance in the brain similar to DM in peripheral lesions. Besides, T2DM and AD often co-exist in several individuals. Recently, several clinical observations have provided significant insights into certain injuries of brain structural and functional (Martinez-Tellez et al., 2005). These impairment in global memory, abstracted reasoning, attention and visual-motor arrangements frequently happened in the diabetic population (Ryan and Geckle, 2000a,b). Moreover, insulin action in the brain was selectively impaired in T2DM, especially in the hippocampus, hypothalamus and cortex (Bruehl et al., 2009). The lesions of insulin action in diverse regions could be attributed to the generation of some factors such as inflammatory mediators, oxidative stress, impaired insulin receptor signaling, Aβ (β-amyloid) plaque and hyper-phosphorylated tau protein.

The medial prefrontal cortex (mPFC), a critical structure in the brain, coordinates the activities of multiple cortical regions, exerts the effects of the ongoing supervisation of information, being critical to daily work (Krueger et al., 2007; Einarsson and Nader, 2012). Accordingly to a current study, memory-related brain circuits in mPFC were impaired in T2DM (Tagougui et al., 2015) and prefrontal atrophy who had been diagnosed (Lee et al., 2013; Moran et al., 2013). A longitudinal neuropsychological study has convincingly shown that patients with damage to PFC exhibit disorders in the higher cognitive functions (Cohen, 2000). Clinical research indicated that the terrible executive function and memory lesion in T2DM correlated predominantly with reduced glucose metabolism in the mPFC and orbital, temporal, and cerebellum regions (Garcia-Casares et al., 2014). Some studies suggested the memory dysfunction occurred in T2DM patients during acute hyperglycemia (Cox et al., 2005). The abnormal change in the insulin signaling pathway might contribute to damage the neuronal structure or function (Zhao and Alkon, 2001) in T2DM, and further disturbed the impulse conduction of the nerve, the transmitter release, information export and so on. Therefore, the mechanisms which triggered neuronal lesions in the mPFC of DM by metabolic syndrome were still a subject that scientists needed to investigate. What’s more important in DM encephalopathy to our studies, BBr whether alleviated the cognitive impairment through modulating insulin signaling transduction and anti-inflammatory pathway in mPFC still require more researches to clarify.

Berberine which derives from Traditional Chinese Medicine has shown its safety and efficiency in human and animals. It is an isoquinoline alkaloid and isolated from *Coptis chinensis* Franch (Teodoro et al., 2013). Recently, BBr has been confirmed to have versatile functions such as anti-inflammation, anti-bacterial, hypoglycemic, cholesterol-lowing, clearance of oxygen radical, anti-cancer and a well-documented effect against dysfunction in various brain nervous systems (Kumar et al., 2015). It has also been verified effective in cerebral ischemia, hypertension, hyperlipemia, mental depression, anxiety and AD (Abd El-Wahab et al., 2013; Kumar et al., 2015). Besides, it exhibited great effects in the face of intestinal mucosal barrier dysfunction in type 2 diabetic rats (Gong et al., 2017).

Fear-stimulating events can persist even over a lifetime and is widely considered to be a highly persistent and durable form of memory (Bergstrom, 2016). The mPFC modulates the expression of contextual fear conditioning (Lisboa et al., 2010). Increasing evidences indicated that contextual fear condition assay could be applied to appraise the lesion in PFC of AD (Moreira et al., 2012; Zelikowsky et al., 2013). However, there are no investigations concerning about whether BBr could suppress DM-induced fear-conditioned cognitive defect. In our study, the contextual fear condition assay was applied to detect the effects of BBr on the fear-conditioned cognitive function of diabetic rats. Moreover, we expounded the probable mechanisms that BBr improved in ordinate inflammation and insulin pathway in mPFC of DM as well.

## Materials and Methods

### Materials

Monoclonal antibodies IR, PI3K, p-NF-κB^Ser536^, p-GSK3β^Ser9^, GSK3β, GLUT3, PKC𝜀, PKCη, BACE-1, APP, glial fibrillary acidic protein (GFAP) and polyclonal antibody Aβ42 were purchased from Abcam. Monoclonal antibodies p-IRS-1^Ser307^, p-AKT^Ser473^, AKT, p-JNK^Thr183/Tyr185^, JNK, p-4E-BP1^Thr37/46^, and NF-κB were obtained from CST. P-PI3K P85^Tyr467^ was purchased from absin. P-IKKβ^Y199^ was purchased from Gene Tex. P-ERK1/2 ^Thr202/Tyr204^ and ERK1/2 were purchased from Millipore. The ladder marker was obtained from Thermo scientific. STZ was purchased from biosci biotechnology Co.; Ltd. ELISA kits were purchased from BOSTER.

### Animals and Treatment

Male Wistar rats weighting about 200g (aged 4–5 weeks) were provided from Beijing vital river laboratory animal technology Co.; Ltd. Animals were housed in SPF circumstance with a 12h light/dark cycle under room temperature. Two rats were raised in a cage and replaced with clean bedding for avoiding humid disgusting environment. Except the NOR (normal group), all of the rats were raised with HSFD (high sugar and high fat diet: 67.5% standard laboratory rat chow, 10% lard, 20% sugar, 2% cholesterol and 0.5% bile salts) for 4 weeks after 2 weeks of adaptive feeding. The diabetic animal model was constructed by injecting 25mg/Kg STZ (streptozocin) through the caudal vein in the HSFD group. One week later, OGTT (oral glucose tolerance test) was applied to appraise diabetic model. Meanwhile, the STZ-treated rats which met the qualification (Post-prandial blood glucose values excess 11.2 mMol/L) were randomly separated into three groups as follows: DM group (diabetes mellitus), BBr group (187.75 mg/Kg/d) and Met group (Metformin, 184mg/Kg/d). The NOR group was given standard rat diet and water *ad libitum*, while the model group was fed with HSFD until being killed. Behavioral test and PET (Positron-Emission Tomography) imaging test were carried out prior to the sacrificed week. All experiments were guided in accordance with the Animal Care and Use Committee affiliated to the Huazhong University of Science and Technology (IACUC Number: S538).

### Preparation of the Prefrontal Cortex Samples

Brains were quickly removed and rinsed in ice-cold saline. Each set of 6 brains was embedded in paraffin after fixing by 4% paraformaldehyde for ICH (immunohistochemistry) and IF (immunofluorescence). After dissecting on a cold plate, the tissues which used for western-blot were flash frozen in liquid nitrogen and preserved -80°C until using.

### Fear Condition

Fear memory was appraised as described previously with minor modification (Peters et al., 2010; Skelton et al., 2011). After 3 days acclimatization, the rats were free inquiry within 120s in a 50 cm × 25 cm × 25 cm fear-chamber before the tone cue was given. On the 1st day, animals were exposed to 3 tones (85 dB, 2.7 kHz, 18 s on/off cycle) followed by 3 tone-foot shock pairings (0.8 mA for 2 s). Another 30 s was used for recovery after shock. For testing the short-memory, the same procedures were executed after 1.5 h. On the following day, the rodents were placed in the box with 3 changed tones (85 dB, 2.7 kHz, 20 s on/off cycle), no shock and 60 s intervals presenting as an extinction test of contextual fear. On the 3rd day, animals were returned to the box with a novel floor and the sides of the box were stuck on color wall paper. After the same 2 min acclimatization, 20 s tones followed with 30 s recovery were presented as same as the 1st day just without 2 s foot shock in this day. Freezing behavior was scored by the experiment equipment (AniLab Software & Instrument). To avoid the presence of olfactory cues, all of the apparatus were thoroughly cleaned with 75% ethanol and wiped with a dry towel in the intervals.

### TNF-α, IL-1, and IL-18 Measurements

Over-expression of diversified pro-inflammation interleukins in the DM had been paid more attention. The supernatant samples of the homogenized tissues were added into the 96 well plates that coated with primary antibody, and disposed by means of the procedures of the manufacturer instructions individually. The O.D. value was monitored at the wavelength of 450 nm after the blue color transforming into yellow immediately. The contents of these cytokines in the sample were calculated according to the standard curve drew from the reference substance reacted in the same system.

### Western Blot

Sixty to eighty micrograms (μg) of total mPFC lysate samples were denatured with the gel-loading buffer at boiling water for 10 min, and followed cooling to electrophorese separation (about 15–20 μL) on 8–10% Tris-glycine polyacrylamide gels. The separated proteins were transferred into PVDF membrane (Millipore, 0.45 um) under the condition of 300 voltage, 120–180 min. BSA (Bovine Serum Albumin) was applied to block and primary antibodies were incubated 12–16 h at 4°C. The reactive protein bands were visualized using the Odyssey imaging system.

### Positron-Emission Tomography (PET) Imaging

*In vivo* PET imaging was performed from the *Trans*-PET^®^ BioCaliburn^®^ 700 (Raycan Technology Co., Ltd, Suzhou, China), and the method was referred to Jin- Min Liu (Liu et al., 2016).

### Prefrontal Cortex Histological Morphology Studies

Some slices were immunochemistry stained with primary monoclonal antibody against GFAP, Aβ42 (Abcam, United States) with the purpose to appraise the activation of astrocyte and the risk of senile plaque formation. Other sections were immunofluorescent stained with the primary antibody against NF-κB (CST, United States). Finally, observation was performed under a fluorescence microscope (Olympus).

### Statistical Analysis

Data were analyzed by Graph Pad Prism 5 software and expressed as mean ±SEM. Statistical significance was assessed by One-way ANOVA following Tukey’s Post Test. *P* values < 0.05 suggested that the level of significances was existed.

## Result

### Effect of BBr on Fear-Related Behaviors in DM Rats

**FIGURE 1:**
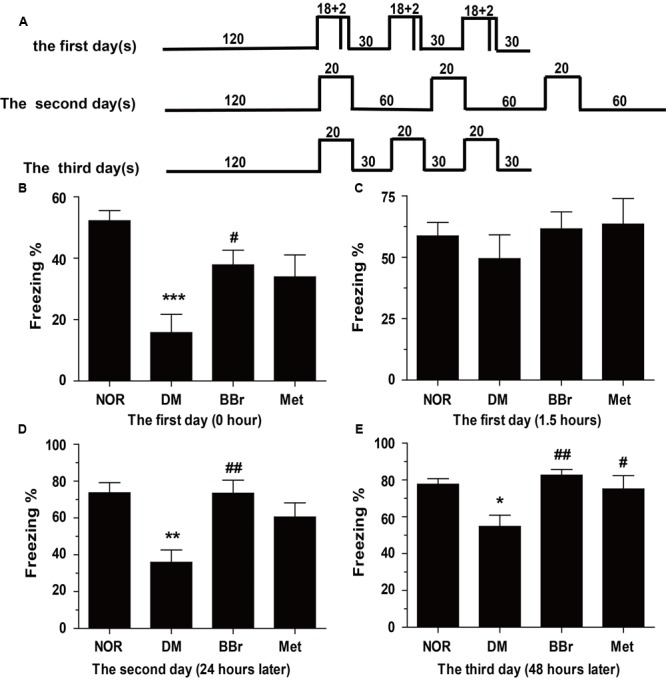
Berberine ameliorates the fear-memory deficit in DM rats. **(A)** Experiment protocol. Rats were exposed to an inescapable chamber and with different cue and contextual fear, and the freezing percent was recorded during the 3 days. **(B)** The freezing percent in the 1st day after 3 days acclimatization. **(C)** The freezing percent in the 1st day after the training trail of 1.5 h later to detect the short-term memory. **(D,E)** The freezing percent in the 2nd **(D)** and 3rd **(E)** day after the training trail to further appraise the long-term memory, and assessed the protection of BBr. ^∗^*P* < 0.05 vs. NOR; ^∗∗^*P* < 0.01 vs. NOR; ^∗∗∗^*P* < 0.001 vs. NOR; **^#^***P* < 0.05 vs. DM; **^##^***P* < 0.01 vs. DM; *n* = 8∼12.

As these characteristics of simply and quickly learning and maintain for a long time even for a lifetime, fear memory is frequently applied as a classical experimental model to study the cerebral mystery which involves in learning and memory especially in rodents (Baldi and Bucherelli, 2015). Compared to NOR group, DM group significantly decreased the percent freezing after accommodating for 24 h (**Figure 1B**: ^∗∗^*P* < 0.01 vs. control), but BBr group increased the percent freezing (**Figure 1B**: ^#^*P* < 0.05 vs. DM) when compared to DM group. After 1.5 h, the same procedures were utilized to detect the percent freezing. As is shown in **Figure 1C**, compare to NOR, these stimulations did not significantly affect the acquisition of the conditioned fear response in DM group. The percent freezing in these groups were all recovered to NOR level. To further explore the influence of the conditioned fear, the changed condition which diminished the 2 s electric stimulus and prolonged the resting time for 60 s (**Figure 1A**) were used to analyze the fear memory response on the following day. Strikingly in **Figure 1D**, NOR and BBr (^##^*P* < 0.01 vs. DM) group exhibited well percent freezing across the trail. On the 3rd day, the condition was back to the criteria of the 1st day, but the 2 s electric stimulus was taken away (**Figure 1A**). Examination of the freezing across the trials (**Figure 1E**) manifested that the NOR and the drug therapy group (^##^*P* < 0.01 vs. DM; ^#^*P* < 0.05 vs. DM) significantly enhanced the freezing percent. The DM rats (^∗∗^*P* < 0.01 vs. NOR) which seemly forgot the electric stimulus exhibited less freezing than NOR after triple recalls.

### BBr Reduces the Insulin Resistance and Regulates Related Protein in the Insulin Signaling Pathway in mPFC of DM Rats

**FIGURE 2:**
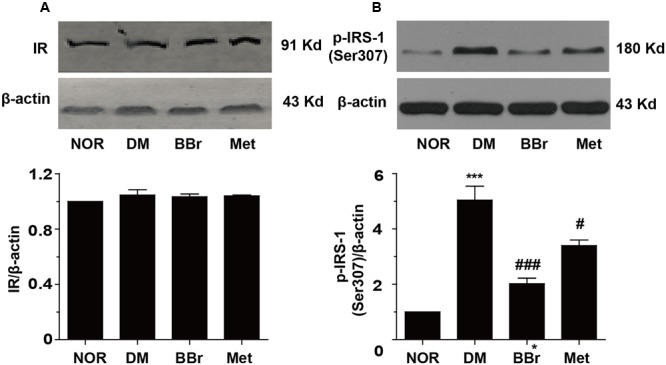
Effect of BBr on insulin resistance in the mPFC of DM rats. **(A)** BBr did not change the expression of insulin receptor, *n* = 4 for western-blot. **(B)** BBr reduced the enhanced expression of p-INS-1^Ser307^, *n* = 3 for western-blot. ^∗∗∗^*P* < 0.001 vs. NOR; **^#^***P* < 0.05 vs. DM. **^###^***P* < 0.001 vs. DM.

**FIGURE 3:**
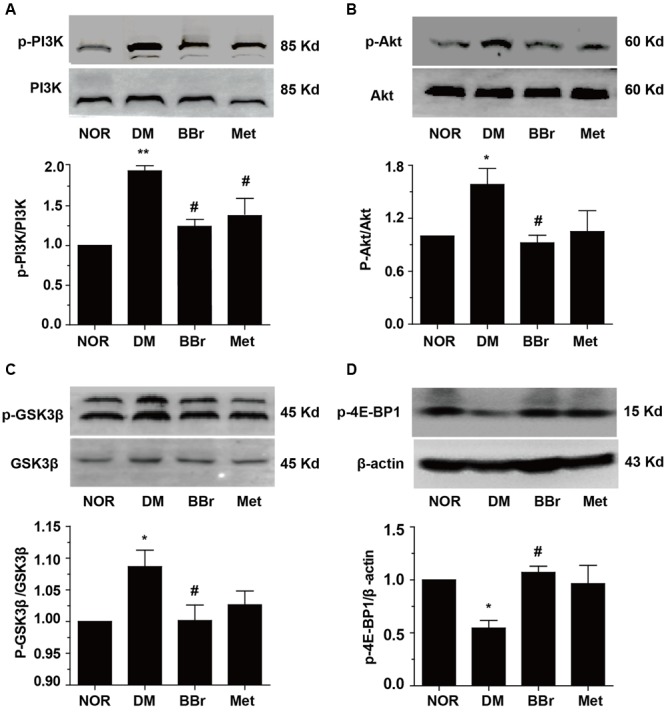
Regulation of PI3K/Akt/mTOR insulin pathway by BBr in the mPFC of DM rats. **(A–D)** BBr reduced the enhanced expressions of p-PI3K ^Tyr467^ **(A)**, p-Akt^Ser473^ **(B)**, p-GSK3β^Ser9^ **(C)**, and p-4E-BP1^Thr37/46^ **(D)** in the mPFC of DM, *n* = 3∼5 for western-blot. ^∗^*P* < 0.05 vs. NOR; ^∗∗^*P* < 0.01 vs. NOR; **^#^***P* < 0.05 vs. DM; **^##^***P* < 0.01 vs. DM.

Some researches indicated that the T2DM patients underlay the diabetes-related cognitive decline, and showed default-mode network in prefrontal cortex (Cui et al., 2015). Insulin signaling pathway was recognized as an association with cognitive function and aging, and disordered signaling pathway might be correlated with memory impaired (Zhao and Alkon, 2001). These dysfunction symptoms were prominent in AD and DM. Obviously, as showed in **Figure 2A**, there were no significant changes on IR (insulin receptor) protein levels in all of our groups. But the expression of p-IRS-1^Ser307^ was significantly increased in DM group (**Figure 2B**), BBr and Met remarkably reduced the increment (^∗∗∗^*P* < 0.001 vs. NOR; ^#^*P* < 0.05 vs. DM; ^###^*P* < 0.001 vs. DM). Next, we detected the phosphorylation of related proteins of insulin signaling pathway and found that most of them were up-regulated in DM group (**Figure 3**). As showed in **Figure 3A**, DM group remarkably increased the expression of p-PI3K when compared to NOR group, but BBr and Met groups obviously reduced its level (^∗∗^*P* < 0.01 vs. NOR; ^#^*P* < 0.05 vs. DM). The same influence occurred in the Akt kinase (**Figure 3B**) that involved in cellular survival pathway. For the BBr and Met groups, they similarly largely attenuated p-Akt^Ser473^ expressions (^∗^*P* < 0.05 vs. NOR; ^#^*P* < 0.05 vs. DM). By measuring the alterations of p-GSK3β^Ser9^, we found that upregulated p-GSK3β^Ser9^ was also inhibited by the isoquinoline alkaloid BBr (^∗^*P* < 0.05 vs. NOR; ^#^*P* < 0.05 vs. DM) when compared with DM group (**Figure 3C**). The biguanide antidiabetic drug Met exhibited a few effects on this protein involved in regulating glycogen synthase. These weird scenes stimulate us to take the activation of mTOR into consideration. Obviously in **Figure 3D**, the substrate protein p-4E-BP1 of mTOR was degraded and BBr inhibited this phenomenon.

### Effect of BBr on Inflammation Signaling Pathway in mPFC of DM Rats

**FIGURE 4:**
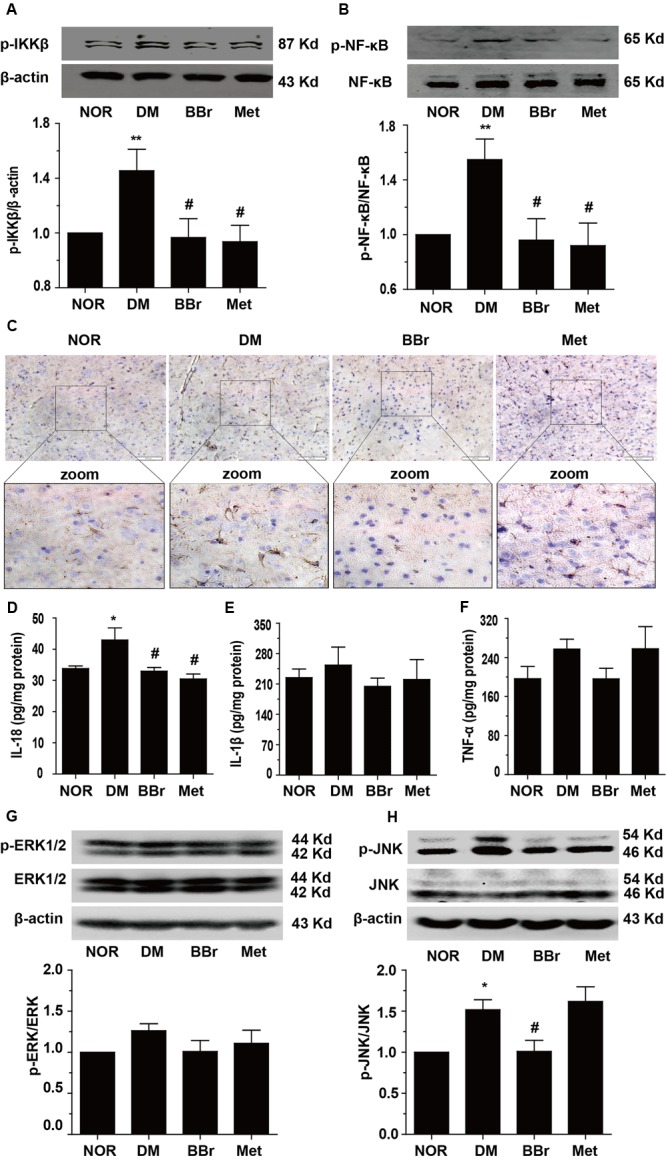
Effect of BBr on inflammation mediators in the mPFC of DM rats. **(A,B)** BBr reduced the increased expression of p-IKK^Y 199^ and p- NF-κB^Ser536^ in the mPFC of DM rats, *n* = 3 for western-blot. **(C)** The immunochemistry showed that the increased expression of GFAP in the mPFC of DM rats was largely inhibited by BBr, *n* = 4 for immunochemistry. **(D–F)** BBr decreased the inflammation cytokine IL-18 **(D)**, but had no effect on IL-1β **(E)**, and TNF-α **(F)** in the mPFC of DM rats, *n* = 8∼10 for ELISA. ^∗^*P* < 0.05 vs. NOR; ^∗∗^*P* < 0.01 vs. NOR; **^#^***P* < 0.05 vs. DM. **(G,H)** BBr reduced the increased expression of p-ERK and p-JNK in the mPFC of DM rats, *n* = 3 for western-blot.

Because a large number of different researches indicated that inflammation could be increased by stress responses which come from peripheral immune and central immune cells released cytokines. Inflammation and its accompanied stress responses are common to neuroplasticity alternation in DM (Sharma et al., 2014; Tian et al., 2016). To determine whether BBr have anti-inflammation effects in the prefrontal cortex, we detected the phosphorylation levels of NF-κB and IKK. As showed in **Figures 4A,B**, the phosphorylation levels of the IKK and NF-κB were greatly augmented in DM group (^∗^*P* < 0.05 vs. NOR; ^∗∗^*P* < 0.01 vs. NOR). In contrast, BBr (^#^*P* < 0.05 vs. DM) strongly reduced these protein′s expression as same as the Met group (^#^*P* < 0.05 vs. DM). For further verifying the inflammation was occurred in astrocyte and was inhibited by BBr, we adopted the immunohistochemistry assay to observe the expression level of GFAP, a marker of astrocyte, in the mPFC of DM rats. Obviously, an urgent increment was showed in DM group when compared to NOR, while these positive expressions were largely decreased by BBr and Met (**Figure 4C**). Next we investigated the inflammatory factors in mPFC and found that BBr did not significantly affect IL-1β and TNF-α except IL-18 (**Figures 4D–F**). IL-18 level (**Figure 4D**) was obviously increased, but a little increment about IL-1β (**Figure 4E**) and TNF-α (**Figure 4F**) also showed in DM when compared with NOR. BBr strikingly suppressed the increment as Met. Based on these observations, we further detected MAPK signaling pathway and found that p-JNK was remarkably enhanced and a few increment about p-ERK in DM (**Figures 4G,H**). These results indicated that BBr reduced the generation of inflammation, the activation of astrocyte and MAPK signaling pathway in mPFC of DM.

### BBr Promotes the Uptake of Glucose in the Brain of DM Rats and Increases the Metabolism of Glucose in Neuron

**FIGURE 5:**
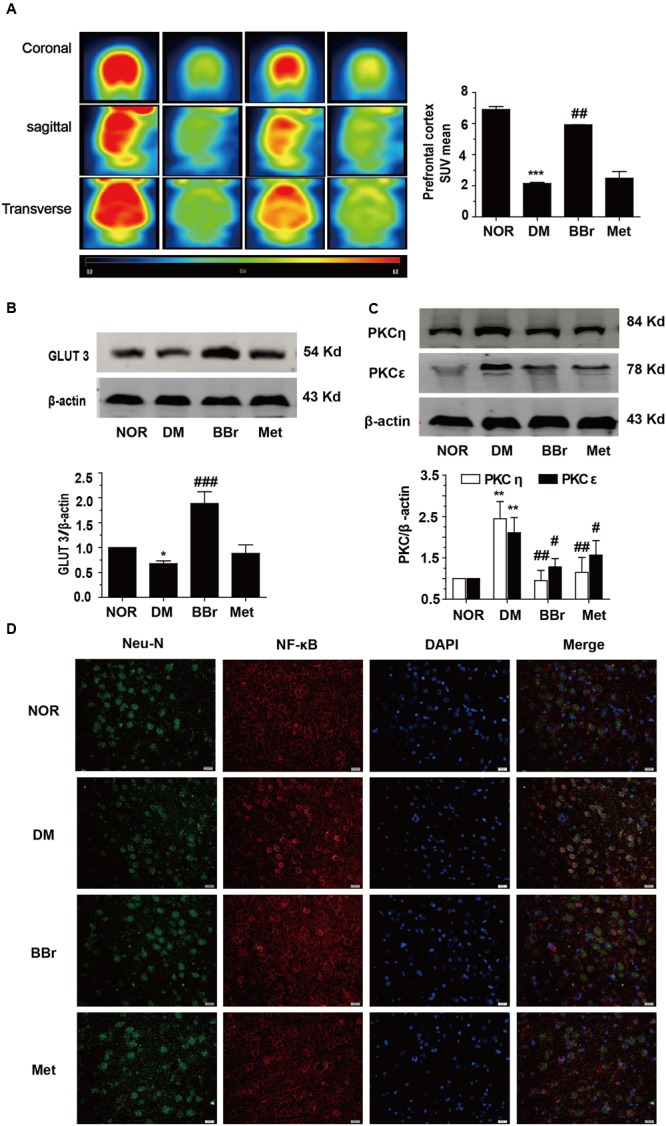
Uptake of glucose in rat brain and expression of GLUT3 in neuron of the mPFC. **(A)** The standard uptake value was described to evaluate the glucose uptake *in vivo* PET imaging. The standard uptake value mean was obviously augmented by BBr, *n* = 3 for PET imaging. **(B)** The down-regulation of the GLUT3 in the mPFC of DM rats was remarkably increased by BBr from Western blot showing, *n* = 3 for western-blot. **(C)** The expression of n-PKC in the mPFC of DM rats was remarkably inhibited by BBr, *n* = 3 for western-blot. **(D)** The translocation of NF-κB in the mPFC of DM rats was remarkably inhibited by BBr, *n* = 3 for immunofluorescence. ^∗^*P* < 0.05 vs. NOR; ^∗∗∗^*P* < 0.001 vs. NOR; **^##^***P* < 0.01 vs. DM.**^###^***P* < 0.001 vs. DM.

The standard uptake value was described to evaluate the glucose uptake *in vivo* PET imaging. The SUV mean was decreased about three times in DM group when compared to NOR, conversely, BBr largely enhanced the value and Met had no effect (**Figure 5A**). For the expression level of GLUT3, it was significantly suppressed in DM group (^∗^*P* < 0.05 vs. NOR) when compared to NOR, but remarkably increased by BBr (^###^*P* < 0.001 vs. DM), and slightly increased by Met (**Figure 5B**). Then we detected the expression of the vital protein kinase C (PKC) which regulated glucose transporter from the cytoplasm to membrane. As showed in **Figure 5C**, the levels of two isoforms PKC𝜀 and PKCη (^∗^*P* < 0.05 vs. NOR;^#^*P* < 0.05 vs. DM; ^##^*P* < 0.01 vs. DM) which came from novel subfamilies co-increased in DM group, whereas their expressions in BBr and Met group were decreased effectively. Meanwhile, we adopted the immunofluorescence assay to observe the translocation of NF-κB from cytoplasm to nucleus. As indicated in **Figure 5D**, the neuron marker Neu-N and transcription factor NF-κB were co-stained in the mPFC. The expression of the NF-κB in the nucleus of the DM neuron was obviously increased. Both BBr and Met remarkably blocked the nuclear translocation of NF-κB (**Figure 5D**).

### Effect of BBr on Aβ42 Production in mPFC of DM Rats

**FIGURE 6:**
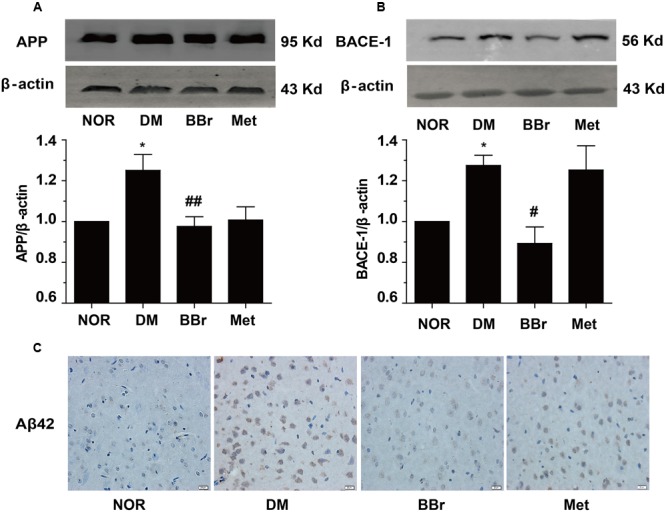
Effect of BBr on amyloid-β formation. **(A,B)** The amyloid precursor protein (APP) and BACE-1 presented in the mPFC of DM rats were remarkably reduced by BBr. However, Met just restrained the APP expression and had no effect on BACE-1, *n* = 3 for western-blot. **(C)** The generation of Aβ42 was effectively inhibited by BBr and Met might play a little effect, *n* = 3 for immunochemistry. **^∗^***P* < 0.05 vs. NOR; **^#^***P* < 0.05 vs. DM; **^##^***P* < 0.01 vs. DM.

Accumulating reports indicated that the dysregulation of the insulin signaling pathway and the presentation of the inflammation favored the Aβ42 production and the formation of senile plaques which were the principal pathological hallmarks of AD (Huang et al., 2017). We next investigated the protein changes involved the Aβ42 production in mPFC of DM rats. As showed in **Figure 6A**, the APP was obviously increased in DM group. BBr strongly reduced the expression level of APP which was induced by diabetes, and Met had the same effects. In addition, we determined the BACE-1 (beta-site amyloid precursor protein cleaving enzyme 1) level (**Figure 6B**), it was surprising that Met had no effect on the enzyme BACE-1 as same as the DM group. This phenomenon was in harmony with other groups (Chen et al., 2009). Contrast to Met group, BBr could almost reverse the status. We found that BBr could suppress the expression of the BACE-1 up to almost two times. Moreover, the production of Aβ42 in mPFC of DM rats was distinctly reduced by BBr (**Figure 6C**). But almost a little reduction was appeared in Met group when compared to DM group.

## Discussion

The cognitive changes induced by diabetes have been acknowledged. Most clinical researches proved the connectivity around the mPFC was impaired in patients with T2DM (Cui et al., 2015). Recent results also suggest that DM could partly affect the dendritic morphology in the occipital cortex, prefrontal cortex and hippocampus (Martinez-Tellez et al., 2005). Aβ accumulation in the brain is the feature of aging and cognitive ability is decreased during the development of pathophysiology, especially in AD and MCI induced by DM (Ma et al., 2015). A number of studies had proved that inflammation promoted the Aβ peptide to aggregate rapidly (Philippens et al., 2016). Moreover, inflammation also disturbed the insulin signaling pathway; conversely, aberrant insulin signaling pathway exacerbated the inflammation production (Nandipati et al., 2016), and led to more Aβ peptide aggregation and impaired cognitive function (Philippens et al., 2016). This study indicated for the first time that monomeric BBr administration modulated aberrant insulin signaling pathway, reduced inflammatory mediators and improved the lesion of cognitive in DM rats.

Met is widely used as a first-line treatment for patients with T2DM. Previous studies demonstrated that Met could rapidly cross the blood-brain barrier (Labuzek et al., 2010) and had several beneficial effects in the brain, such as anti-inflammatory (Wang et al., 2016) and neuroprotective effects (Gupta et al., 2011). Meanwhile, Met could alleviate the impairment of cognitive function in db/db mice (Li et al., 2012; Chen et al., 2016). Many clinic trails were also shown that long-term treatment with Met could reduce the risk of cognitive decline (Guo et al., 2014; Ng et al., 2014). So Met is one of the best candidates for the positive control in the DM-induced cognitive impaired model.

Here, we used the contextual fear condition assay to test BBr on the improvement of the cognitive function. On the 1st day, we found the freezing percent in DM group was decreased (**Figure 1B**) when compared to NOR. BBr group seemingly quickly remembered the influence of stimulation and significantly increased the freezing percent (**Figure 1B**). After 1.5-h intervals, the percent freezing in DM groups was almost recovered to NOR level (**Figure 1C**). The same results also emerged in the drug groups in our study. This might indicate the retrieval of information in short-term memory, no matter DM group and the treatment groups, almost all recovered up to normal level. Besides, the results probably also suggested that BBr group dealt with problems more quickly when compared to DM group (**Figures 1B,C**). On the next day, the freezing percent in DM group was decreased once again than in the 1st day when detection was executed after 1.5 h, but no significant changes were found in BBr (**Figure 1D**). These observations indicated that BBr accelerated the reinforcement of the information. Meanwhile, BBr still significantly augmented the freezing percent when compared to DM after 24 h. The same results occurred in the 3rd day, and Met also ameliorated the fear memory defect. It may imply that long-term memory, rather than short-term memory is impaired in DM and BBr could effectively inhibit the cognitive impairment. Moreover, BBr seemed to reduce cognitive impairment which caused by DM earlier than Met (**Figures 1D,E**).

Desensitized insulin receptor signaling was involved in both AD and DM. In most of the previous literature, it had been demonstrated that venenous Aβ oligomer caused partial loss of insulin receptor in dendritic spine mainly in aged primary hippocampus neurons, with increased distribution of insulin receptor in the cell soma but no alternations in absolute IR subunit levels, rendering insulin receptor non-responsive in the AD brain (Zhao et al., 2008). In the present study, our results also showed that no distinctive changes of the IR protein levels in mPFC of the four groups (**Figure 2A**). Of course, this result needs more experiments to classify whether IR internalization was generated. Phospho-Ser307 in IRS-1 blocks the interaction between the IRS-1 PTB domain and insulin receptor, which might weaken the coupling between the two proteins (McEwen and Reagan, 2004) and lead to insulin resistance. Our results distinctly revealed the increased p-IRS-1^ser307^ in DM group was significantly reduced by BBr. The condition of insulin resistance in the mPFC of DM rats was also inhibited by Met (**Figure 2B**). Additionally, the ROS generation which mediated by HG-induced also activated the aberrantly PI3K/Akt signaling pathway (Lee and Han, 2010). Chronically excessive activation of Akt also caused the development of Tau pathological and Aβ42 generation under insulin resistant situation in db/db and HSFD mouse brains (Kim et al., 2015). In our study, activated PI3K/ Akt signaling pathway of DM-induced was remarkably inhibited in BBr and Met groups (**Figures 3A,B**). Similarly, the excessive activation or overexpression of GSK3β could trigger a series of pathological alterations, most of which are hallmarks of T2DM and AD (Gao et al., 2011). Additionally, overproduction of APP also obviously activated the GSK3β in the hippocampus of APP-V717I amyloid transgenic mice (Terwel et al., 2008). In our research, the increased p-GSK3β^Ser9^ protein was significantly rectified by BBr in the mPFC of DM as well (**Figure 3C**). In additionally, the activation of mTOR signaling pathway was involved in inflammation transduction (Xiao et al., 2017) and facilitation of neurodegeneration in AD (Wang et al., 2014). Obviously, the decreased mTOR substrate p-4E-BP1 (Thr37/46) in our experiment (**Figure 3D**) indicated that the PI3K/Akt/mTOR signaling was activated. This is coherent to other group (Karki et al., 2016). In summary, these results ascertained that activated PI3K/Akt/mTOR in DM group might accelerate the generation of Aβ (**Figures 3A–D**).

There is a close relationship between the impairment of cognitive function and the activation of inflammatory pathways (Li Y.B. et al., 2016). The transcription factor NF-κB was a key protein involved in cellular responses to stimulations such as oxidation stress, ultraviolet irradiation, bacterial, cytokines and so on. The release of the NF-κB is an initiation of the degradation of IκB proteins via activation of a kinase IKK. Our previous researches indicated that BBr attenuates the production of inflammatory mediators through suppression of TLR4 (toll-like receptor 4)/NF-κB signaling in intestine tissue of diabetic rats. The present work was performed to study the anti-inflammation effect of BBr in mPFC of DM rats. The activation of n-PKC also attributed to the upregulation of NF-κB. We speculated that BBr not only reduced the phosphorylation levels of IKKβ and NF-κB (**Figures 4A,B**), but also suppressed the upregulation of n-PKC (**Figure 5C**). Moreover, as showed in **Figure 4C**, the expression of FGAP in DM group was inhibited by BBr as well as Met. Meanwhile, the increased protein levels of pro-inflammation IL-18 (**Figure 4D**) in DM group were significantly decreased by BBr in the supernatant fluids of the tissue homogenate, but few effects on IL-1β and TNF-a (**Figures 4E,F**) were found. In additionally, the activation of MAPK signaling pathway was involved in inflammation transduction (Zhang et al., 2017) and facilitation of neurodegeneration in AD (Kirouac et al., 2017). Our results also ascertained that activated JNK (**Figure 4H**) was showed in DM group and BBr decreased the increment. The sustained neuroprotective effects of BBr observed in the present study is consistent with the research about protecting against neuronal damage via suppression of inflammation in traumatic brain injury (Chen et al., 2014). Taken together, our findings suggested that BBr developed its neuroprotective function against cognitive deficits in DM rats via inhibiting the production of inflammation in astrocyte.

Then we focus on the uptake and metabolism of glucose in the mPFC of DM brain. *In vivo* PET imaging is a common method to detect the uptake of glucose in brain. The impairment of glucose uptake in the brain of T2DM aggravated the pathological alteration. This phenomenon also emerges in our research. In **Figure 5A**, the shiny red color which represented the uptake of glucose in NOR was completely disappeared in the DM. The therapeutic drug BBr obviously recovered the uptake of glucose. Met had a slightly increment. Of note, the high density of GLUT3 in neurons was pivotal in the energy supplement and neuronal activities. Moreover, the impaired glucose uptake/metabolism was also inversely correlated to the densities of NFTs (Neurofibrillary tangles) (Liu et al., 2008). In our study, the decreased GLUT3 was presented in the mPFC of DM rats (**Figure 5B**). Traditional Chinese medicine BBr could largely enhance GLUT3 expression and reverse the lesion. Emerging evidence indicated that protein kinase C epsilon (PKC𝜀) participated in insulin-regulated trafficking of the glucose transporter GLUT4 in adipocytes (Tsuchiya et al., 2013). Then, we determined the expression level of PKC and found that these conclusions were more or less discrepant with our discoveries. As show in our results (**Figure 5C**), both of two novel Ca^2+^-insensitive isoforms of PKC were activated under insulin resistance. The distinct scene might be a negative feedback regulation in the mPFC, and BBr effectively blunt the increase extent of n-PKC (novel-PKC) in DM rats as well as Met. This was coherent to some researches (Brownlee, 2001; Giorgi et al., 2010) that more than one PKC isoform in DM were activated by hyperglycemia, since many PKC isoforms were DAG-dependent style. Nevertheless some reports indicated that PKC𝜀 activation was required for recognition memory in the rats’ hippocampus (Zisopoulou et al., 2013). Meanwhile, the translocation of NF-κB was also increased in neuron of mPFC of DM rats. This might indicate that the neuron in DM group had some capacities to resist the generation of damages. But, the exact molecular mechanism needs further investigation. Overall, related proteins were activated in mPFC of DM in the insulin signaling pathway except GLUT3 in neuron, this scene might involve in some other cells activation but not neuron, just as the astrocyte. These results indicate that BBr not only facilitates glucose metabolism in mPFC, but also promotes the uptake of glucose, even in the DM rats under cognitive impairment.

It is well known that Aβ42 is the primary pathogenic factor in AD. Growing evidences indicated that DM was one of the upper most risk factors for developing AD and accelerated Aβ42 pathological changes (Moran et al., 2015). APP is best known as the precursor molecule whose proteolysis generates Aβ. It was reasonable to observe the incremental levels of APP and BACE-1 in mPFC of DM (**Figures 6A,B**) because their increases also appeared in monkey with DM (Okabayashi et al., 2015). The administration of BBr can reduce the incremental levels of APP and BACE-1 in mPFC of DM (**Figures 6A,B**) and clean the oligomeric toxic protein Aβ42 (**Figure 6C**). However, it remains unknown how BBr promote the reduction of APP levels. In this regard, it still needs great efforts to examine the underlying mechanisms about the effect on transcriptional inhibition or translation degeneration. As the mentioned above, insulin sensitization agent Met reduced the APP level, but the enzyme BACE-1 level was largely augmented. This co-action might accelerate the Aβ42 production as in DM and this discovery had been reported before (Chen et al., 2009). There are maybe some reasons, such as rapid degradation or some other kinase activities were enhanced, ascribe to the weaker function in Met group.

## Conclusion

**FIGURE 7:**
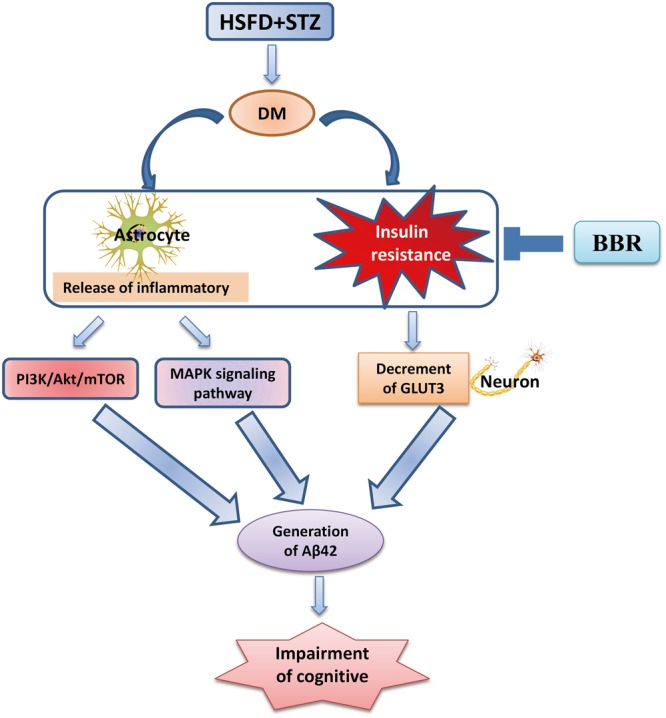
Possible mechanism of BBr for ameliorating the cognitive impairment in DM rats.

Our present findings (**Figure 7**) suggest that BBr not only modulated the aberrant inflammation responses, but also regulated some in ordinate proteins of insulin signaling pathway in the mPFC of DM rats. Moreover, it promoted the uptake of glucose in the brain of DM rats and increased the metabolism of glucose in neuron. These results probably more clearly elucidate that BBr exhibits the importance of multi-targets in the mPFC of DM. Moreover, these apparently protective actions of BBr prevent diabetes-associated cognitive decline in DM rats.

## Ethics Statement

The study protocol was in agreement with Animal Care and Use Committee affiliated to the Huazhong University of Science and Technology.

## Author Contributions

QC and RM performed most of the experiments. NW, XZ, and CS participated in data analyses. JG and KF contributed to the discussion of the manuscript. JL, DW, and DY helped to the preformation of the experiments and collected the samples. KW and JC participated in its design and coordination. All authors read and approved the final manuscript.

## Conflict of Interest Statement

The authors declare that the research was conducted in the absence of any commercial or financial relationships that could be construed as a potential conflict of interest.
